# Retrospective Validation of Clinical Decision Support Tools for Predicting Effectiveness Outcomes in Inflammatory Bowel Disease Patients Treated with Vedolizumab

**DOI:** 10.3390/pharmaceutics18010015

**Published:** 2025-12-22

**Authors:** Paul A. G. de Klaver, Amber M. H. van Woerkens, Hedwig M. A. D’Agnolo, Marieke J. Pierik, Luc J. J. Derijks

**Affiliations:** 1 Department of Clinical Pharmacy and Pharmacology, Máxima Medical Center, 5504 DB Veldhoven, The Netherlands; a.m.h.vanwoerkens@students.uu.nl (A.M.H.v.W.);; 2 Department of Pharmaceutical Sciences, Utrecht University, 3584 CG Utrecht, The Netherlands; 3 Department of Gastroenterology and Hepatology, Máxima Medical Center, 5504 DB Veldhoven, The Netherlands; hedwig.dagnolo@mmc.nl; 4 Department of Gastroenterology and Hepatology, Maastricht University Medical Center, 6229 HX Maastricht, The Netherlands; m.pierik@mumc.nl; 5 School of Nutrition and Translational Research in Metabolism (NUTRIM), Maastricht University, 6229 ER Maastricht, The Netherlands; 6 Department of Clinical Pharmacy and Toxicology, Maastricht University Medical Center, 6229 HX Maastricht, The Netherlands

**Keywords:** vedolizumab, inflammatory bowel disease, Crohn’s disease, ulcerative colitis, clinical decision support tool, remission, drug survival

## Abstract

**Background/Objectives**: Two clinical decision support tools (CDSTs) have been developed to predict treatment effectiveness of vedolizumab in Crohn’s disease (CD) and ulcerative colitis (UC). This study aimed to validate the CDSTs with real-world data from a Dutch teaching hospital. **Methods**: Patients with CD or UC treated with vedolizumab between October 2014 and July 2023 were included. IBD patients treated with ustekinumab were included to study the specificity of the CDSTs. The primary outcomes were rates of clinical remission (CREM), biochemical remission (BioREM), composite (either clinical or biochemical) remission (CompREM) and corticosteroid-free clinical remission (CSF-CREM) at week 14, 30 and 54. **Results**: 32 CD patients and 41 UC patients treated with vedolizumab were included, as well as 28 CD patients treated with ustekinumab and 41 UC patients treated with vedolizumab. Among UC patients treated with vedolizumab, rates of CREM, CompREM and CSF-CREM at week 54 were statistically significantly higher in the group with high probability of response compared to the group with low + intermediate probability of response (CREM 33.3% vs. 81.8% *p* = 0.003; CompREM 33.3% vs. 86.4% *p* < 0.001; CSF-CREM 22.2% vs. 77.3% *p* = 0.001). For both CD patients treated with vedolizumab and CD patients treated with ustekinumab, no statistically significant differences were found between low + intermediate and high probability groups for all outcomes. **Conclusions**: The CDST for UC is able to predict various effectiveness outcomes for treatment with vedolizumab and can therefore help in the selection of optimal treatment for patients with UC. For CD patients treated with vedolizumab, the CDST could not predict effectiveness outcomes.

## 1. Introduction

Inflammatory bowel disease (IBD) refers to a group of chronic inflammatory conditions that affect the gastrointestinal (GI) tract including ulcerative colitis (UC) and Crohn’s disease (CD) [1]. In recent decades, more and more different therapeutic options for achieving and maintaining remission in IBD have become available, including monoclonal antibodies [2]. While multiple therapeutic options are available for the treatment of IBD, unfortunately a proportion of patients will experience treatment failure or loss of response during treatment [3]. In combination with the increasing number of monoclonal antibodies available for the treatment of IBD, it is of great value to be able to predict treatment response before initiating therapy. This is beneficial for the patients to receive effective treatment as quickly as possible, but also for society, considering monoclonal antibodies often are very expensive.

One such costly monoclonal antibody used in the treatment of IBD is vedolizumab. Vedolizumab is a humanized monoclonal antibody that binds selectively to the α_4_β_7_ integrin expressed on the surface of gut-homing T lymphocytes. This drug blocks the interaction between these cells with the mucosal addressin cell adhesion molecule-1 (MAdCAM-1). As a result, extravasation of T lymphocytes into the gastrointestinal submucosa is inhibited, which in turn suppresses the subsequent inflammatory response [4]. Vedolizumab is indicated for the treatment of adult patients with moderately to severely active CD or UC who had an inadequate response or were intolerant to conventional therapy or anti-TNF-alpha [5].

Recently, Dulai et al. developed two clinical decision support tools (CDSTs) to determine response probability of vedolizumab in patients with CD and UC. These CDSTs were built using data from the GEMINI 1 and 2 trials and validated using data from the US VICTORY consortium [6,7]. Because the CDSTs were developed with data from registration trials of vedolizumab, it is important to further validate these models with real world data as conditions may differ. Several research groups already conducted a validation study using real world data with varying results: a Korean study showed that the CDST had acceptable accuracy in patients with UC; however, the utility of the CDST for patients with CD was limited. In contrast, a French study found that the CDST for patients with CD was able to predict clinical remission [8,9].

Due to the limited research and varying results in several real-world settings, it is important to further assess the applicability of the CDSTs for vedolizumab in both patients with CD and UC in the Netherlands. Therefore, this study includes the retrospective validation of two CDSTs for predicting effectiveness outcomes in IBD patients treated with vedolizumab with real-world data from a Dutch teaching hospital. Additionally, the specificity of both CDSTs for vedolizumab is investigated by also including IBD patients treated with ustekinumab. We tested for specificity, since this was not investigated in the original CDST development studies, and parameters included in the CDSTs seem aspecific for the drug vedolizumab.

## 2. Materials and Methods

### 2.1. Patients

In this study, a retrospective analysis was conducted with data from adult IBD patients treated in Máxima Medical Center (MMC), the Netherlands, with either intravenous vedolizumab or ustekinumab between October 2014 and July 2023. Patients who lacked necessary baseline data for calculating the probability score or follow-up data for determining outcomes were excluded. Additionally, patients who received ustekinumab for other indications than IBD (mainly psoriasis) and those who only received the initial intravenous induction dose of ustekinumab were excluded. Patients who were treated with both vedolizumab and ustekinumab during the study period have been included twice, receiving two separate scores: a score based on the baseline data at the start of vedolizumab and another based on the baseline data at the start of ustekinumab.

### 2.2. Vedolizumab Clinical Decision Support Tools for CD and UC

Dulai et al. developed two different CDSTs to identify patients with CD and UC who respond to vedolizumab treatment. Data from GEMINI 1 and 2 trials were used to identify factors associated with clinical, steroid-free and durable remission. The clinical decision support tools were developed based on these factors. Dulai et al. also defined cut-off points to determine whether patients have low, intermediate or high probability of response [6,7].

Both CDSTs use different items to calculate a probability score and have different cut-off points. For included patients diagnosed with CD, the probability score was calculated using the following five items:

- No prior bowel surgery (+2 points);
- No prior TNF inhibitor therapy (+3 points);
- No prior fistulising disease (+2 points);
- Baseline albumin (+0.4 points per unit [g/L]);
- Baseline C-reactive protein (CRP) (−0.5 points if 3.0–10.0 mg/L, −3.0 points if >10 mg/L).

CD patients with a score of ≤13, >13 to ≤19 and >19 were classified as having low, intermediate or high probability of response to vedolizumab treatment, respectively [6].

For included patients diagnosed with UC, the probability score was calculated using the following four items:

- Disease duration ≥ 2 years (+3 points);
- No prior TNF inhibitor therapy (+3 points);
- Baseline endoscopy moderate activity (+2 points);
- Baseline albumin concentration (+0.65 points per unit [g/L]).

UC patients with scores of ≤26, >26 to ≤32 and >32 points were classified as having low, intermediate or high probability of response to vedolizumab treatment, respectively [7].

### 2.3. Data Collection

Patient demographics, clinical characteristics and laboratory results were collected from the electronic medical records of MMC using CTcue version 4.15.2 (CTcue B.V., Amsterdam, The Netherlands) to ensure that the data was pseudo-anonymized. This data included gender, age, BMI, smoking habits, disease duration, disease location/extent, history of perianal disease, history of fistulising disease, history of bowel surgery, previous anti-TNF exposure, corticosteroid use at initiation and disease activity at initiation. Laboratory results included serum albumin (g/L), CRP (mg/L) and fecal calprotectin (mg/kg) at baseline. The Montreal classification was used to record disease location for CD (L1–L3) and disease extent for CU (E1–E3).

### 2.4. Outcomes

The primary outcomes of this study were rates of clinical, biochemical, composite (either clinical or biochemical) and corticosteroid-free clinical remission. These outcomes were assessed at week 14 ± 2 weeks (post-induction), week 30 ± 8 weeks (6 months maintenance) and week 54 ± 8 weeks (12 months maintenance). Clinical remission (CREM) was defined as a Harvey–Bradshaw Index (HBI) ≤ 3 for CD, a Simple Clinical Colitis Activity Index (SCCAI) ≤ 2 for UC or physician global assessment (PGA) described as ‘remission’ or ‘no symptoms’ in the medical record. Biochemical remission (BioREM) was defined as C-reactive protein (CRP) < 5 mg/L or fecal calprotectin (fCalpro) < 250 mg/kg, and corticosteroid-free clinical remission (CSF-CREM) was defined the same as CREM without the concomitant use of corticosteroids. Composite remission (CompREM) was defined as either CREM or BioREM or both.

Secondary outcomes firstly included drug survival (drug persistence). The end of the treatment was defined as the date of the last administration of vedolizumab, plus the interval between doses. It was not possible to determine the end of treatment for ustekinumab with certainty, given that ustekinumab is administered subcutaneously by the patient at home. Secondly, the incidence of a reduced dosing interval (<8 weeks) and the incidence of bowel resection was assessed at week 54. Patients who received bowel resection were considered to have stopped treatment with vedolizumab or ustekinumab and to have failed to achieve any form of remission. The correlation between baseline serum albumin level and drug survival at week 54 was assessed since baseline albumin level forms a major part of the CDST scores. Finally, the accuracy of the CDSTs for predicting drug survival was assessed. Patients with missing data around week 14, week 30 or week 54 were excluded from the evaluation of remission outcomes of the week(s) with missing data. Ustekinumab was used as a marker for CDST specificity for vedolizumab in this study.

### 2.5. Statistical Analyses

Normality of continuous variables was assessed using skewness and kurtosis. A variable was considered to have a normal distribution if skewness and kurtosis were between −1 and +1. Additionally, normality was graphically assessed using histograms and quantile-quantile plots. Normal continuous variables were reported as mean with standard deviation and non-normal continuous variables were reported as median with interquartile range (IQR). Categorical variables were reported as numbers and percentages.

For each treatment group, differences in the rates of patients achieving outcomes between the probability groups were analyzed using Fisher’s exact test. To further evaluate drug survival, Kaplan–Meier curves were created in which the time until discontinuation of vedolizumab was shown for all probability groups. The differences between these curves were assessed using the log-rank test. Sensitivity and specificity were assessed for a cut-off of 19 points for the CDST of CD and for a cut-off of 32 points for the CDST of UC to predict drug survival. These are the cut-off points between having intermediate or high probability of response according to the CDSTs. The accuracy of both CDSTs was assessed by receiver operating characteristic (ROC) curve analyses, after which the area under the curve (AUC) was determined. Finally, the correlation between baseline serum albumin and drug survival was assessed using the Mann–Whitney U-test to evaluate whether baseline serum albumin is a predictor of drug survival. Boxplots were created to illustrate the differences in baseline serum albumin between patients with and without drug survival.

If the number of patients in a specific probability group was limited, sensitivity analyses on merged groups was performed. All *p*-values were two-sided and considered statistically significant when *p* < 0.05. Statistical analyses and data visualizations were performed using IBM SPSS statistics version 29 (IBM Corp, Armonk, NY, USA) and R (version 4.5.0; R Core Team, 2025).

## 3. Results

### 3.1. Patient Characteristics

**Figure 1:**
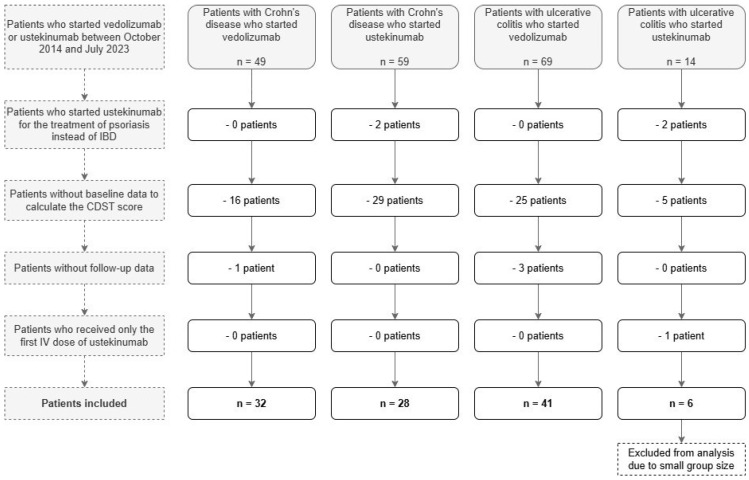
Study flowchart with inclusion and exclusion criteria. IBD: inflammatory bowel disease; CDST: clinical decision support tool. Arrows indicate the flow of patients, minus signs (-) indicate the number of patients excluded in this part of the flow chart.

The study flowchart is shown in Figure 1. Among 118 patients with IBD who received vedolizumab and 73 patients with IBD who received ustekinumab, 75 patients were excluded because of missing baseline data to calculate the probability of response score. Four patients with ustekinumab treatment were excluded because they started ustekinumab for psoriasis instead of IBD, and one patient was excluded because the patient only received the first intravenous (IV) induction dose of ustekinumab. Four patients were excluded because of missing follow-up data. After exclusion, six patients with UC treated with ustekinumab remained. Due to this limited number, these patients were also excluded since no meaningful statistical analysis could be performed on this group.

As a result, 101 patients were included: 32 with CD treated with vedolizumab, 28 with CD treated with ustekinumab and 41 with UC treated with vedolizumab. These patients were classified into low, intermediate and high probability of response groups based on their CDST-scores. This resulted in the following number of people in each probability group: 8 patients with low probability (1 with CD and vedolizumab, 5 with CD and ustekinumab, 2 with UC and vedolizumab), 55 patients with intermediate probability (21 with CD and vedolizumab, 18 with CD and ustekinumab, 16 with UC and vedolizumab) and 38 patients with high probability (10 with CD and vedolizumab, 5 with CD and ustekinumab, 23 with UC and vedolizumab). Due to the limited number of patients with low probability of response, a sensitivity analysis was performed to evaluate the impact of merging these groups with the intermediate probability groups. Based on the results, which showed minimal changes in significance, the low and intermediate probability of response groups were merged for all subsequent analyses.

In Table 1 baseline patient characteristics are shown. As none of the continuous variables were found to have a normal distribution, they were reported as median with IQR. Among included patients, baseline characteristics were similar between the treatment groups, except for baseline fCalpro in which the median was lower in CD patients treated with vedolizumab (365.5 [149.8–1407.3]) and CD patients treated with ustekinumab (751.5 [442.5–2432.2]) in comparison to UC patients treated with vedolizumab (2230.0 [477.5–3515.0]). Baseline fCalpro was unknown for 10 CD patients treated with vedolizumab, for 18 CD patients treated with ustekinumab and for 20 UC patients treated with vedolizumab. There were relatively more females among patients with CD (68.8% with vedolizumab and 78.6% with ustekinumab) than among patients with UC (48.8%).

### 3.2. Clinical and Biochemical Outcomes

**Figure 2:**
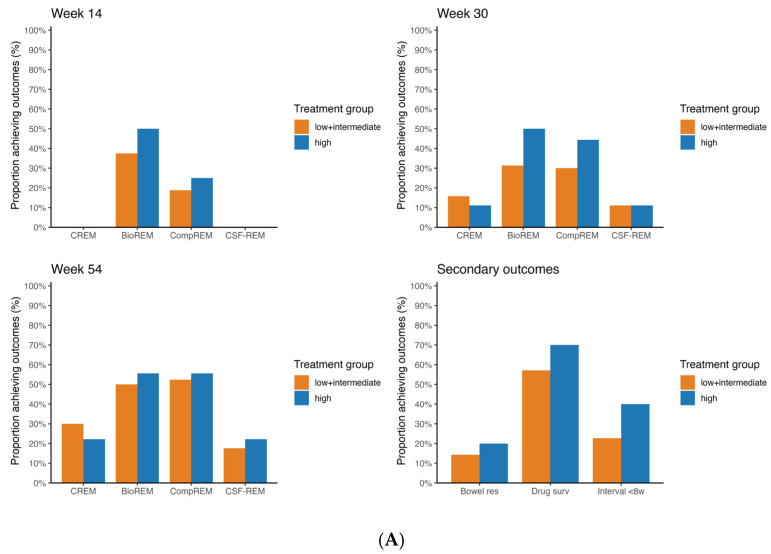

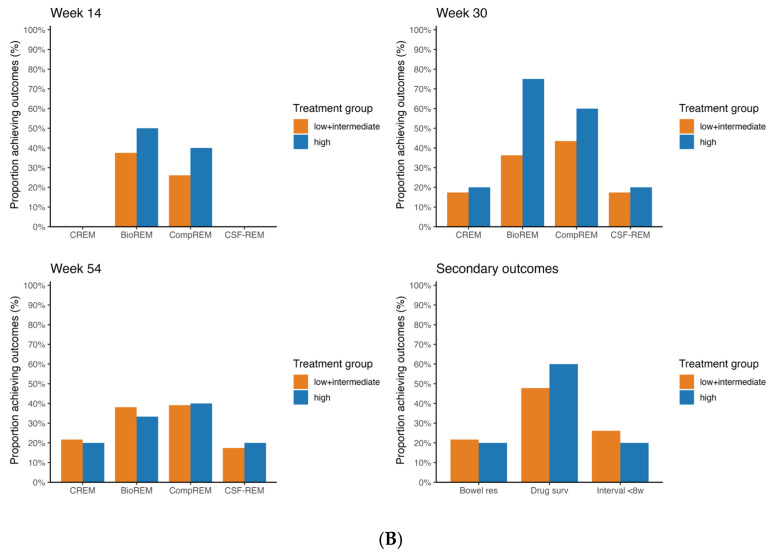
(**A**) Achievement of effectiveness outcomes in CD patients treated with vedolizumab proportion achieving primary outcomes are shown for week 14, week 30 and week 54. Secondary outcomes are shown for week 54. The orange and blue bars represent low + intermediate and high probability groups, respectively. CREM: Rates of clinical remission; BioREM: Biochemical remission; CompREM: Composite clinical or biochemical remission; CSF-CREM: Corticosteroid-free clinical remission. (**B**) Achievement of effectiveness outcomes in CD patients treated with ustekinumab. The proportion achieving primary outcomes are shown for week 14, week 30 and week 54. Secondary outcomes are shown for week 54. The orange and blue bars represent low + intermediate and high probability groups, respectively. CREM: Rates of clinical remission; BioREM: Biochemical remission; CompREM: Composite clinical or biochemical remission; CSF-CREM: Corticosteroid-free clinical remission.

**Figure 3:**
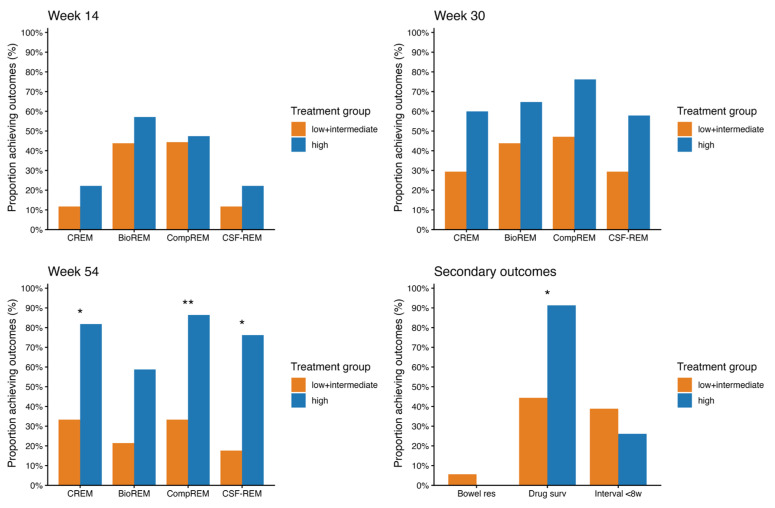
Achievement of effectiveness outcomes in UC patients treated with vedolizumab. The proportion achieving primary outcomes are shown for week 14, week 30 and week 54. Secondary outcomes are shown for week 54. * *p* < 0.01, ** *p* < 0.001. The orange and blue bars represent low + intermediate and high probability groups, respectively. CREM: Rates of clinical remission; BioREM: Biochemical remission; CompREM: Composite clinical or biochemical remission; CSF-CREM: Corticosteroid-free clinical remission.

In the assessment of clinical and biochemical outcomes, patients were excluded from evaluation of certain outcomes when there were missing data. Figure 2 and Figure 3 show the rates of CREM, BioREM, CSF-CREM and CompREM at week 14, 30 and 54 along with the number of patients that were included at each outcome.

Figure 2A shows the rates of CREM, BioREM, CompREM and CSF-CREM at week 14, week 30 and week 54 for CD patients treated with vedolizumab (week 54 CREM 30.0% vs. 22.2% *p* = 1.000; BioREM 50.0% vs. 55.6% *p* = 1.000; CompREM 52.4% vs. 55.6% *p* = 1.000; CSF-CREM 17.6% vs. 22.2% *p* = 1.000). In this group, there were no incremental trends in the rates of CREM and CSF-CREM at week 14, week 30 and week 54. The high probability group showed similar rates of CREM and CSF-CREM compared to the low + intermediate group (*p* = 1.000). In week 14, no patients had clinical remission. Regarding BioREM and CompREM, there were increasing trends visible (mainly at week 30: BioREM 31.3% vs. 50.0% *p* = 0.624; CompREM 30.0% vs. 44.4% *p* = 0.783), but the differences were not statistically significant. Figure 2B shows the same rates but for CD patients treated with ustekinumab. Similarly, there were no statistically significant differences in the rates of CREM, BioREM, CompREM and CSF-CREM between the low + intermediate probability group and the high probability group at all three timepoints (week 54 CREM 16.7% vs. 20.0% *p* = 0.57; BioREM 31.3% vs. 33.3% *p* = 0.685; CompREM 33.3% vs. 40.0% *p* = 0.648; CSF-CREM 11.1% vs. 20.0% *p* = 0.228). In both CD patients treated with vedolizumab and those treated with ustekinumab, sensitivity analysis showed no differences in statistical significance.

Among UC patients treated with vedolizumab, rates of CREM, CompREM and CSF-CREM at week 54 were statistically significantly higher in the group with high probability of response compared to the group with low + intermediate probability of response (CREM 33.3% vs. 81.8% *p* = 0.003; CompREM 33.3% vs. 86.4% *p* < 0.001; CSF-CREM 22.2% vs. 77.3% *p* = 0.001) (Figure 3). The rate of BioREM at week 54 also showed an increasing trend, but this difference was not statistically significant (21.4% vs. 58.8% *p* = 0.067). At week 14 and week 30, rates of all outcomes were higher for patients with high probability of response compared to patients with low + intermediate probability of response, but the differences were not statistically significant. Since both UC patients treated with vedolizumab with low probability had CREM, BioREM, CompREM and CSF-CREM at, respectively, week 14 and week 30, combining these patients with the intermediate probability group slightly decreased differences in significance. The difference in BioREM at week 54 and the differences in CREM, CompREM and CSF-CREM at week 30 were statistically significant when comparing only the intermediate group with the high probability group.

### 3.3. Secondary Outcomes

Figure 2 and Figure 3 show the rates of bowel resection, drug survival and patients with dosing interval < 8 weeks at week 54 for patients with CD and UC.

**Figure 4:**
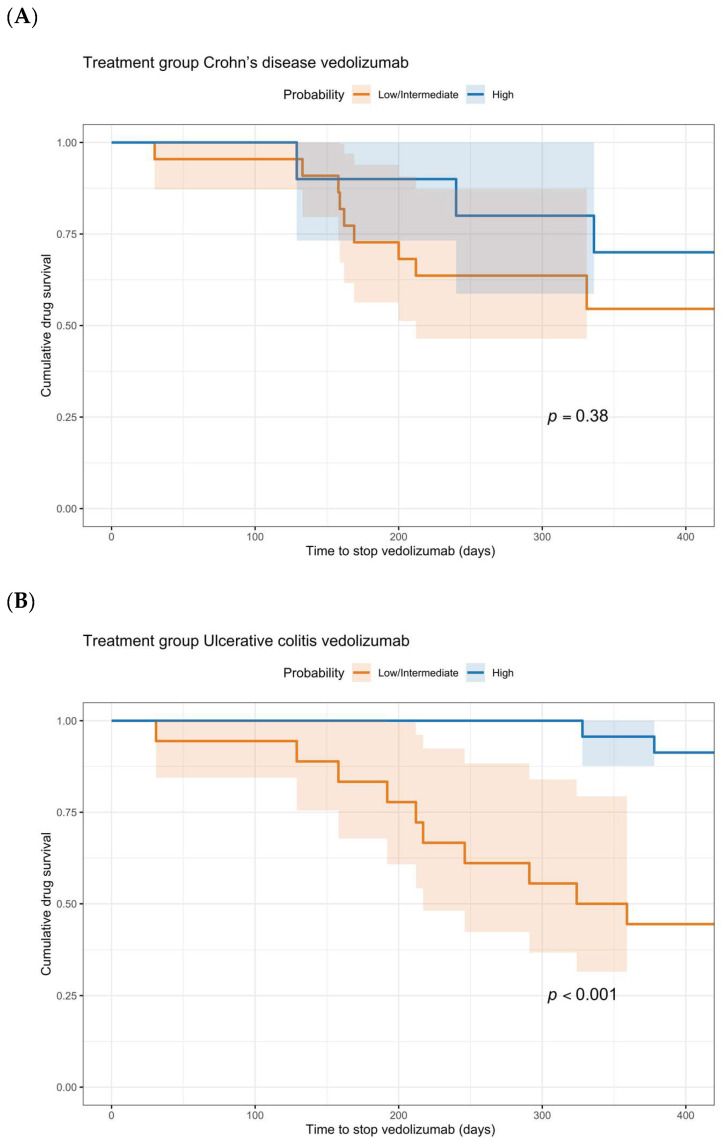
Cumulative rates of drug survival for patients with CD (**A**) and UC (**B**) treated with vedolizumab. The orange and blue curves represent low + intermediate and high probability groups, respectively. Kaplan–Meier survival curves are presented with 95% confidence intervals. Confidence intervals are not displayed beyond approximately 300 days of follow-up because the number of patients at risk was insufficient to allow reliable estimation. The survival curves themselves are shown until the maximum follow-up, but confidence intervals naturally cease once data support is lacking.

For patients with CD treated with vedolizumab, there were no statistically significant differences between the low + intermediate probability group and the high probability group for all three outcomes (week 54 persistance of treatment 57.1% vs. 70.0% *p* = 0.430). The same applies for CD patients treated with ustekinumab (week 54 persistance of treatment 47.8% vs. 60.0% *p* = 0.321). For patients with UC treated with vedolizumab, rate of drug survival in the group with low + intermediate probability of response differed statistically significant from the group with high probability of response (44.4% vs. 91.3% *p* < 0.001). Regarding the rates of bowel resection and of a reduced dosing interval, no statistically significant differences were found. The Kaplan–Meier curves for drug survival are shown in Figure 4. These curves show a statistically significant difference between low + intermediate probability and high probability for UC patients treated with vedolizumab (*p* < 0.001). In CD patients treated with vedolizumab, no statistically significant difference was found (*p* = 0.379).

**Figure 5:**
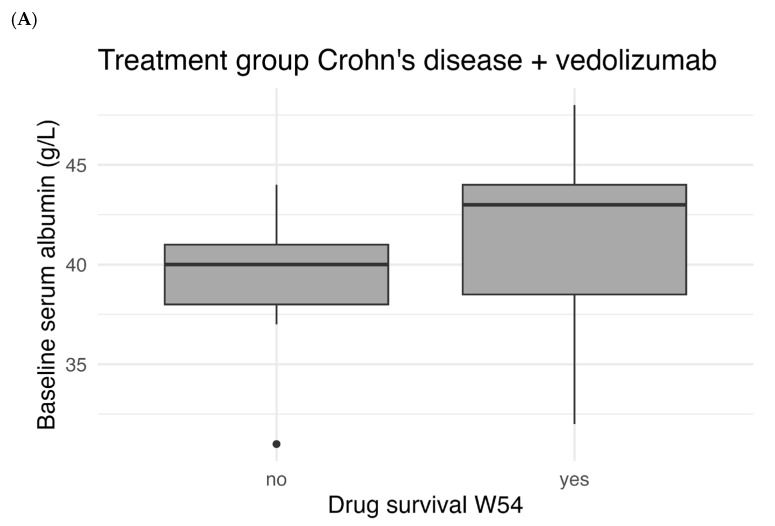

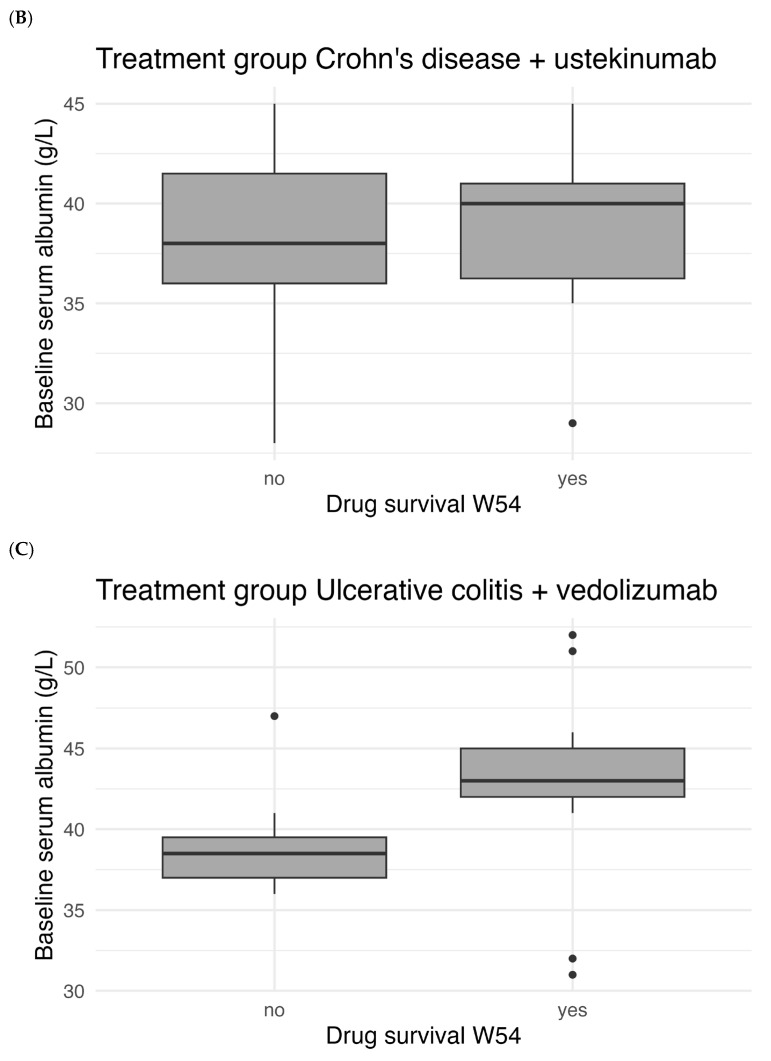
Correlation between baseline serum albumin (g/L) and drug survival at week 54 (W54) for CD patients treated with vedolizumab (**A**), ustekinumab (**B**) and UC patients treated with vedolizumab (**C**). The boxes represent the area between the first and third percentile (and median shown in the box). The whiskers extend to the smallest and largest values within 1.5× interquartile range. The dots indicate outliers outside 1.5× interquartile range.

Figure 5 shows the correlation between baseline serum albumin levels and drug survival. There was a statistically significant difference in median baseline albumin between patients with and without W54 drug survival for UC patients treated with vedolizumab (*p* < 0.001). For patients with CD there was no statistically significant difference for both treatment with vedolizumab (*p* = 0.106) and ustekinumab (*p* = 0.712).

### 3.4. Sensitivity and Specificity

For the CDST of CD, a cut-off value of 19 points had a specificity of 76.9% and a sensitivity of 36.8% in predicting drug survival at week 54 in vedolizumab treated patients. The AUC for predicting drug survival in CD patients treated with vedolizumab was 0.636 (95%CI 0.439–0.832). For ustekinumab-treated CD patients, the AUC for predicting drug survival was 0.495 (95%CI 0.273–0.716) and a cut-off of 19 points had a specificity of 85.7% and a sensitivity of 21.4%.

The CDST of UC discriminated drug survival at week 54 with an AUC of 0.843 (95%CI 0.718–0.968). A cut-off value of 32 points had a specificity of 83.3% and a sensitivity of 72.4% in predicting drug survival at week 54. ROC curves are shown in the Supplementary Materials.

## 4. Discussion

In this study, we assessed the value of two CDSTs in predicting achievement of effectiveness outcomes in patients with CD or UC treated with vedolizumab. We also studied the specificity of both CDSTs by including IBD patients treated with ustekinumab. The cohort of ustekinumab-treated UC patients was too small for testing specificity of the CDST and was therefore not included in analysis.

For patients with UC, the CDST predicted CREM, CompREM, CSF-CREM and drug survival of vedolizumab at week 54. These findings are consistent with the results from a previous Korean real-world validation study [8]. The Korean study found a significantly higher rate of clinical remission at week 54 in patients with high probability (46.9%) than in patients with intermediate probability (29.8%). Our study also found a significant difference in clinical remission at week 54 between the groups with low + intermediate and high probability, but clinical remission rates were higher in our study (33.3% vs. 81,8%) compared to the Korean study. An explanation for this difference could be a difference in rates of disease extent. In the Korean study, 51.1% of the UC patients treated with vedolizumab had extensive colitis (E3) compared to 26.8% in this study. Scarozza et al. found that patients with left-sided colitis (E2) are more likely to achieve clinical remission than patients with extensive colitis following vedolizumab treatment [10]. Consistent with our findings, a study by Dulai et al. found a higher rate of clinical remission at week 52 in vedolizumab treated UC patients with high probability of response (39.7%) compared to those with intermediate probability of response (15.2%) [11]. Again, these rates were substantially lower than in our study. An explanation for this difference could be that the inclusion criteria for disease activity were more stringent, e.g., having a total Mayo score of 6 to 12. Therefore, CRP and fCalpro were higher and baseline albumin was lower in the study of Dulai et al. compared to our study. The differences in CRP and fCalpro could also account for the non-significance in BioREM between the UC patient groups treated with vedolizumab in our study.

In CD patients treated with vedolizumab, no statistically significant differences in any form of remission were found between low + intermediate and high probability groups in our study. Regarding clinical remission and CSF-clinical remission, we even found an opposite trend: slightly higher rates of clinical remission and CSF-clinical remission were found in CD patients with intermediate probability than in patients with high probability. The Korean study found a similar trend: 30.4% of CD patients with intermediate probability of response had clinical remission at week 54 compared to 0.0% of CD patients with high probability of response. In contrast to our study, Alric et al. reported statistically significant differences between CD patients with intermediate probability and CD patients with high probability treated with vedolizumab [9]. The authors reported that 80% of the patients with high probability achieved clinical remission at week 48 compared to 38.5% of the patients with intermediate probability. The difference in clinical remission rates could be due to the difference in definition of clinical remission: Alric et al. defined clinical remission as having a HBI ≤ 4, whereas in our study clinical remission was defined by the GPA (‘remission’ or ‘no symptoms’ in the report) or by having a HBI ≤ 3. Possibly, patients in the study of Alric et al. achieved clinical remission more easily than in our study. Regarding drug survival, Alric et al. reported a significant difference between CD patients with intermediate probability (51.9%) and CD patients with high probability (80.0%). In our study there was also a trend visible in rate of drug survival for CD patients treated with vedolizumab: 70.0% of patients with high probability of response had drug survival at week 54 compared to 54.5% of patients with intermediate probability of response. However, this difference was not statistically significant in our study. The AUC of 0.636 (95%CI 0.439–0.832) also showed a positive, but not statistically significant, trend for predicting drug survival at week 54. For CD patients treated with ustekinumab, similar results were found in all rates of remission compared to CD patients treated with vedolizumab. The AUC of 0.495 (95%CI 0.273–0.716) showed that the CDST is not able to discriminate drug survival for CD patients treated with ustekinumab in contrast to CD patients treated with vedolizumab, where a higher AUC for predicting drug survival was found.

This study has some limitations, the first being its retrospective design. More than a third of patients (41.4%) were excluded because of missing baseline data (mainly albumin levels) or because of missing follow-up information. This substantially reduced the number of included patients, could have led to selection bias and could limit generalizability. Eventually, there was 1 CD patient and 2 UC patients treated with vedolizumab with low probability of response. The small size of the groups with low probability limited analysis of these groups, so they were merged with the groups with intermediate probability after conducting sensitivity analysis. In patients with CD, this had no effect on the significance of results, but in patients with UC some results were no longer statistically significant since both UC patients with low probability achieved remission. Due to the limited number of patients, UC patients treated with ustekinumab could not be included in analysis (n = 6). As a consequence, it could not be determined to what extent the CDST for UC is specific for vedolizumab. Another limitation was that determination of clinical remission was dependent on recorded information, where an objective disease activity score was often lacking. This meant that determination of clinical remission mainly relied on the PGA, which is more subjective. This subjectivity may have introduced variability into the classification of clinical remission.

This study has also several strengths. First, this validation study used real-world data with a follow-up period of 1 year. Vedolizumab appeared to have a slower onset of action compared to anti-TNF therapies, so a longer follow-up period is of great value for validation of the CDSTs [12]. For patients with UC, significant differences were indeed only found at week 54. Second, multiple effectiveness outcomes were evaluated, including clinical and biochemical remission, as well as drug survival. Recently, Alsoud et al. found that the CDSTs more accurately predict drug survival compared to clinical remission, and Choon et al. found that drug survival correlates with more objective measures of biochemical and endoscopic activity [13,14]. Besides that, drug survival can be well determined retrospectively since vedolizumab is administered via infusion at the hospital. Therefore, we would definitely recommend using drug survival as an outcome score for future research.

## 5. Conclusions

This study shows that the CDSTs are able to predict various effectiveness outcomes and can therefore help in the selection of optimal treatment for patients with UC but not for patients with CD in the Dutch population. Real-world data studies with larger numbers of patients are necessary to further validate the CDSTs in IBD patients, especially for CD patients and low probability of response groups.

## Figures and Tables

**Table 1:** Baseline characteristics of included patients. Continuous variables are shown as median (IQR) and categorical as n (%). CD: Crohn’s disease; UC: Ulcerative colitis; n: number; IQR: interquartile range; anti-TNF: anti-tumor necrosis factor; fCalpro: fecal calprotectin.

	CD + Vedolizumab	CD + Ustekinumab	UC + Vedolizumab
	*n* = 32	*n* = 28	*n* = 41
Female, n (%)	22 (68.8)	22 (78.6)	20 (48.8)
Age (years), median (IQR)	44.0 (34.0–57.0)	45.0 (33.3–59.8)	49.0 (26.5–59.5)
BMI (kg/m^2^), median (IQR)	24.4 (21.9–28.1)	24.1 (21.0–28.7)	25.4 (22.8–28.2)
Active smoking, n (%)	5 (15.6)	6 (21.4)	5 (12.2)
Disease duration (years), median (IQR)	11.6 (3.7–18.1)	6.9 (3.0–14.7)	4.7 (2.3–14.4)
Disease location, n (%)			
Ileal (L1)	8 (25.0)	6 (21.4)	-
Colonic (L2)	11 (34.4)	9 (32.1)	-
Ileocolonic (L3)	13 (40.6)	13 (46.4)	-
Disease extent, n (%)			
Proctitis (E1)	-	-	2 (4.9)
Left-sided colitis (E2)	-	-	28 (68.3)
Extensive colitis (E3)	-	-	11 (26.8)
History of perianal disease, n (%)	8 (25.0)	10 (35.7)	
Prior anti-TNF exposure, n (%)	28 (87.5)	28 (100.0)	33 (80.5)
Prior bowel surgery, n (%)	10 (31.)	12 (42.9)	0 (0.0)
Corticosteroid use at initiation, n (%)	19 (59.4)	18 (64.3)	23 (56.1)
Baseline serum albumin (g/L), median (IQR)	40.5 (38.0–43.8)	38.5 (36.0–41.8)	43.0 (39.0–44.0)
Baseline CRP (mg/L), median (IQR)	5.0 (1.9–25.5)	13.0 (2.8–28.5)	4.4 (2.2–9.2)
Baseline fCalpro (mg/kg), median (IQR)	365.5 (149.8–1407.3)	751.5 (442.5–2432.3)	2230.0 (477.5–3515.0)
Missing	*n* = 18	*n* = 10	*n* = 20
Baseline fecal calprotectin > 250 mg/kg, n (%)	10 (66.7)	15 (83.3)	23 (85.2)
Missing	*n* = 17	*n* = 10	*n* = 14

## Data Availability

The datasets generated in and/or analyzed in the current study are available from the corresponding author on request.

## Supplementary Materials

The following supporting information can be downloaded at: https://www.mdpi.com/article/10.3390/pharmaceutics18010015/s1.

## Abbreviations

The following abbreviations are used in this manuscript:

AUC	Area under the curve
BioREM	Biochemical remission
CD	Crohn’s disease
CDST	Clinical decision support tool
CompREM	Composite clinical or biochemical remission
CREM	Rates of clinical remission
CRP	C-reactive protein
CSF-CREM	Corticosteroid-free clinical remission
fCalpro	Fecal calprotectin
GI	Gastro-intestinal
HBI	Harvey-Bradshaw index
IBD	Inflammatory bowel disease
IQR	Interquartile range
IRB/IEC	Institutional review board/Independent ethics committee
IV	intravenous
MAdCAM-1	Mucosal addressin cell adhesion molecule-1
MMC	Máxima Medical Center
PGA	Physician global assessment
ROC	Receiver operating characteristic
SCCAI	Simple clinical colitis activity index
TNF-alpha	Tumor necrosis factor alpha
UC	Ulcerative colitis
WMO	Wet medisch-wetenschappelijk onderzoek met mensen

## References

[1] Morton H., Pedley K.C., Stewart R.J.C., Coad J. (2020). Inflammatory Bowel Disease: Are Symptoms and Diet Linked?. Nutrients.

[2] Klang E., Barash Y., Soffer S., Shachar E., Lahat A. (2021). Trends in inflammatory bowel disease treatment in the past two decades-a high-level text mining analysis of PubMed publications. United Eur. Gastroenterol. J..

[3] Hazel K., O’Connor A. (2020). Emerging treatments for inflammatory bowel disease. Ther. Adv. Chronic Dis..

[4] Wyant T., Fedyk E., Abhyankar B. (2016). An Overview of the Mechanism of Action of the Monoclonal Antibody Vedolizumab. J. Crohn’s Colitis.

[5] European Crohn’s and Colitis Organisation, Interventions Vedolizumab. https://www.e-guide.ecco-ibd.eu/interventions-therapeutic/vedolizumab.

[6] Dulai P.S., Boland B.S., Singh S., Chaudrey K., Koliani-Pace J.L., Kochhar G., Parikh M.P., Shmidt E., Hartke J., Chilukuri P. (2018). Development and Validation of a Scoring System to Predict Outcomes of Vedolizumab Treatment in Patients with Crohn’s Disease. Gastroenterology.

[7] Dulai P.S., Singh S., Vande Casteele N., Meserve J., Winters A., Chablaney S., Aniwan S., Shashi P., Kochhar G., Weiss A. (2020). Development and Validation of Clinical Scoring Tool to Predict Outcomes of Treatment with Vedolizumab in Patients With Ulcerative Colitis. Clin. Gastroenterol. Hepatol..

[8] Kim K., Park J.J., Yoon H., Lee J., Kim K.O., Kim E.S., Kim S.Y., Boo S.J., Jung Y., Yoo J.H. (2024). Application of clinical decision support tools for predicting outcomes with vedolizumab therapy in patients with inflammatory bowel disease: A KASID multicentre study. Aliment. Pharmacol. Ther..

[9] Alric H., Amiot A., Kirchgesner J., Treton X., Allez M., Bouhnik Y., Beaugerie L., Carbonnel F., Meyer A. (2022). Vedolizumab Clinical Decision Support Tool Predicts Efficacy of Vedolizumab but Not Ustekinumab in Refractory Crohn’s Disease. Inflamm. Bowel Dis..

[10] Scarozza P., Marafini I., Laudisi F., Troncone E., Schmitt H., Lenti M.V., Costa S., Rocchetti I., De Cristofaro E., Salvatori S. (2020). Extent of Mucosal Inflammation in Ulcerative Colitis Influences the Clinical Remission Induced by Vedolizumab. J. Clin. Med..

[11] Dulai P.S., Wong E.C.L., Reinisch W., Colombel J.F., Marshall J.K., Narula N. (2022). Decision Support Tool Identifies Ulcerative Colitis Patients Most Likely to Achieve Remission with Vedolizumab vs Adalimumab. Inflamm. Bowel Dis..

[12] Vasudevan A., Gibson P.R., van Langenberg D.R. (2017). Time to clinical response and remission for therapeutics in inflammatory bowel diseases: What should the clinician expect, what should patients be told?. World J. Gastroenterol..

[13] Alsoud D., Sabino J., Ferrante M., Verstockt B., Vermeire S. (2025). Calibration, Clinical Utility, and Specificity of Clinical Decision Support Tools in Inflammatory Bowel Disease. Clin. Gastroenterol. Hepatol..

[14] Choon X.Y.W.C., Sharma E., Dart R., Gecse K.B., Mawdsley J., Anderson S.H., Ray S., Irving P., Samaan M.A. Prognostic value of the vedolizumab Crohn’s disease clinical decision support tool in a real-world cohort. Proceedings of the United European Gastroenterology Week.

