# Supplementary figures and images for: Retrospective Validation of Clinical Decision Support Tools for Predicting Effectiveness Outcomes in Inflammatory Bowel Disease Patients Treated with Vedolizumab
Source: Pharmaceutics. 2025 Dec 22;18(1):15. doi: 10.3390/pharmaceutics18010015 (PMC12845096; doi:10.3390/pharmaceutics18010015)

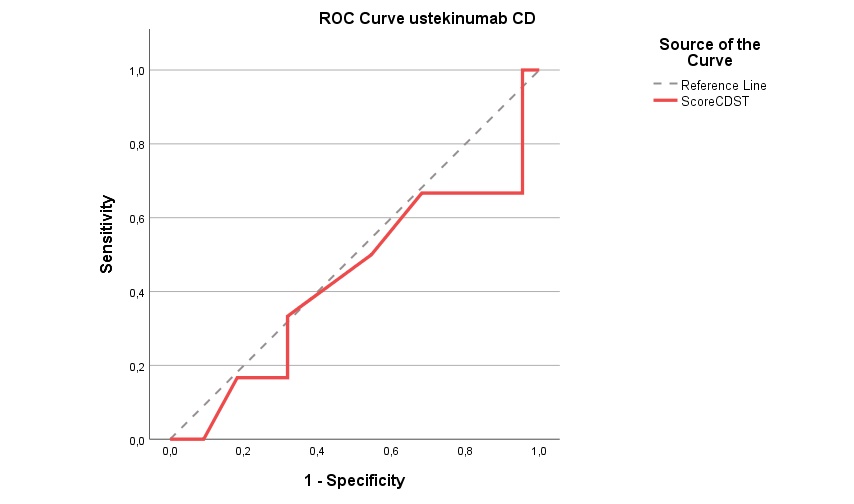

Supplement: Supplementary file 1 [file pharmaceutics-18-00015-s001.zip › pharmaceutics-3997305-supplementary/ROCcurveCDustekinumab.jpg]

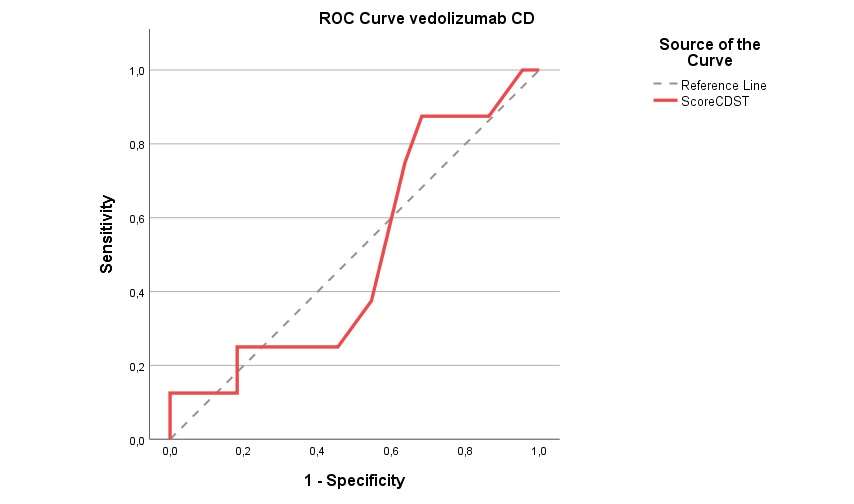

Supplement: Supplementary file 1 [file pharmaceutics-18-00015-s001.zip › pharmaceutics-3997305-supplementary/ROCcurveCDvedolizumab.jpg]

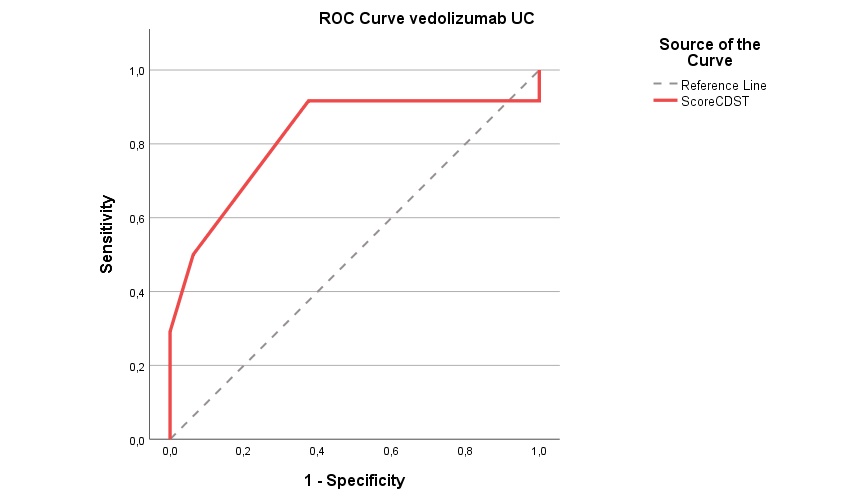

Supplement: Supplementary file 1 [file pharmaceutics-18-00015-s001.zip › pharmaceutics-3997305-supplementary/ROCcurveUCvedolizumab.jpg]

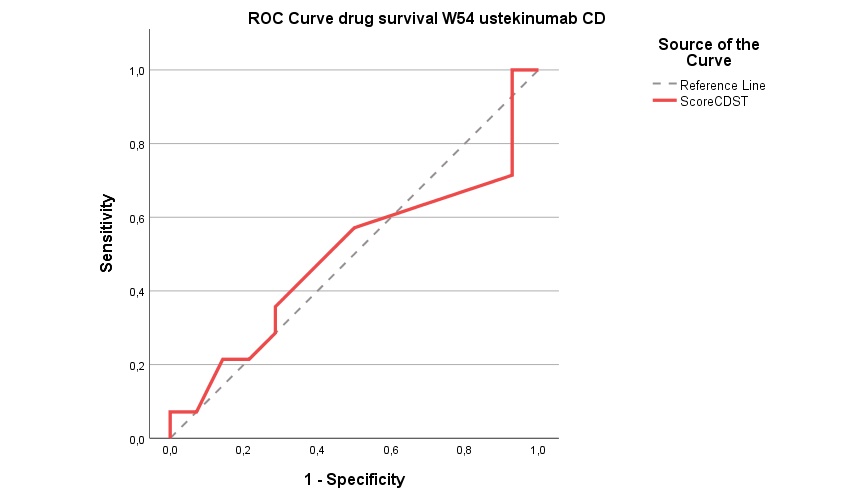

Supplement: Supplementary file 1 [file pharmaceutics-18-00015-s001.zip › pharmaceutics-3997305-supplementary/ROCdrugsurvival ustekinumabCD.jpg]

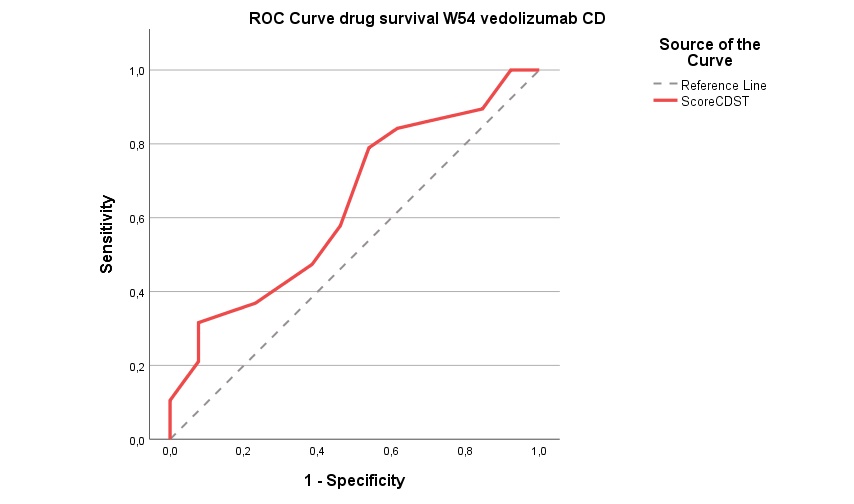

Supplement: Supplementary file 1 [file pharmaceutics-18-00015-s001.zip › pharmaceutics-3997305-supplementary/ROCdrugsurvival vedolizumabCD.jpg]

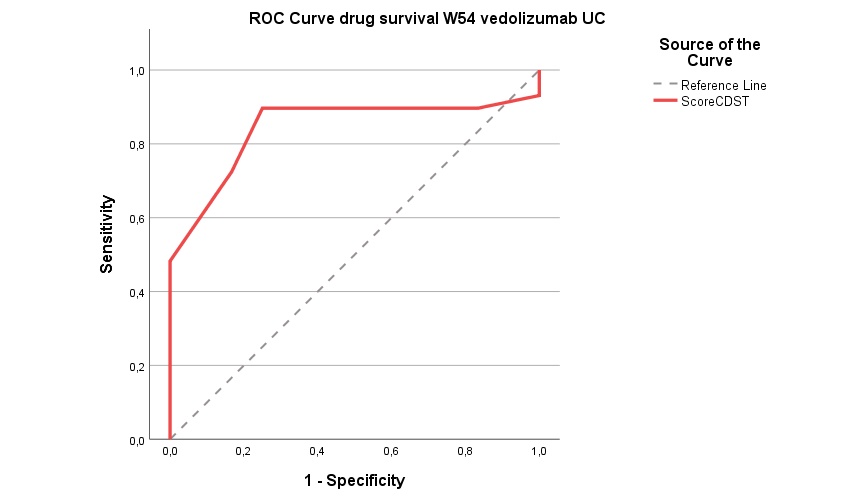

Supplement: Supplementary file 1 [file pharmaceutics-18-00015-s001.zip › pharmaceutics-3997305-supplementary/ROCdrugsurvival vedolizumabUC.jpg]
